# Supplementary material for: Dynamic targeting enables domain-general inhibitory control over action and thought by the prefrontal cortex
Source: Nat Commun. 2022 Jan 12;13:274. doi: 10.1038/s41467-021-27926-w (PMC8755760; doi:10.1038/s41467-021-27926-w)
Supplement: Supplementary file 1 — Supplementary Information [file 41467_2021_27926_MOESM1_ESM.pdf]

Supplementary Information

Supplementary Note 1: Domain-general region overlap with the Cole-Anticevic brain-wide networks

To characterise our domain-general activation results in relation to large-scale brain networks, we used a publicly available Cole-Anticevic brain-wide network partition (CAB-NP)[1]. The CAB-NP was derived from resting-state fMRI data across the whole brain and used the Louvain community detection algorithm to assign parcellated cortical regions[2] into 12 functional networks. We used the Connectome Workbench software[3] to overlay our activations over the CAB-NP to estimate the parcel and network locations of our activation clusters. We did so separately for our within-subjects Stop > Go & No-Think > Think conjunction clusters (Supplementary Figure 1), meta-analytic Stop > Go & No-Think > Think conjunction clusters (Supplementary Figure 2), and the conjunction of the within-subjects conjunction & meta-analysis conjunction clusters (Supplementary Figure 3).

Specifically, we mapped our volumetric activation clusters onto a group average of 1200 subjects' surface template provided by the Connectome Workbench. We used '-volume-to-surface-mapping' command with trilinear volume interpolation. We then overlayed the resulting shape files of our activation results onto the CAB-NP and obtained the corresponding parcel and network locations. For each parcel, we also identified its global variability coefficient (GVC), between-network variability coefficient (BVC) and network partition deviation numbers from Cocuzza at al.[4] discovery and replication data sets. In Supplementary Tables 1-3, we present the identified parcels for each of our activation clusters (from Table 1 in the main text), their network assignment and centre X, Y, Z coordinates, and GVC, BVC and deviation values averaged across the Cocuzza et al.[4] discovery and replication data sets.

Supplementary Table 1. Within-subjects Stop > Go & No-Think > Think conjunction clusters (from Table 1 in the main text) and their estimated parcels, networks, centre X, Y, Z coordinates, global variability coefficients (GVC), between-network variability coefficients (BVC), network partition deviation numbers, and parcel descriptions.

Parcel	Network	X	Y	Z	GVC	BVC	deviation	Parcel description
**1. R VLPFC & Insula**								
254	Frontoparietal	53	19	13	0.3469	0.3486	0.6563	R_Area_44
258	Cingular-Opercular	53	11	13	0.3534	0.3553	0.0000	R_Rostral_Area_6
288	Cingular-Opercular	39	16	6	0.3717	0.3721	0.8907	R_Frontal_Opercular_Area_4
289	Cingular-Opercular	38	13	1	0.3663	0.3691	0.1172	R_Middle_Insular_Area
291	Frontoparietal	33	26	-4	0.3289	0.3299	0.3204	R_Anterior_Ventral_Insular_Area
349	Cingular-Opercular	38	28	4	0.3714	0.3724	0.0078	R_Area_Frontal_Opercular
**Mean**					0.3564	0.3579	0.3321	
Cingular-Opercular					0.3657	0.3672	0.2539	
Frontoparietal					0.3379	0.3393	0.4884	
**2. Right IPL**								
205	Cingular-Opercular	63	-37	27	0.3789	0.3818	0.1797	R_PeriSylvian_Language_Area
208	Posterior Multimodal	57	-45	22	0.3609	0.3620	0.3282	R_Superior_Temporal_Visual_Area
328	Cingular-Opercular	60	-30	38	0.3443	0.3446	0.0000	R_Area_PF_Complex
329	Frontoparietal	51	-50	45	0.3351	0.3361	0.2501	R_Area_PFm_Complex
**Mean**					0.3548	0.3561	0.1895	
**3. Right SMA**								
206	Language	8	19	64	0.3794	0.3819	0.1954	R_Superior_Frontal_Language_Area
224	Cingular-Opercular	20	7	66	0.3463	0.3477	0.5079	R_Area_6m_anterior
278	Frontoparietal	20	25	57	0.3812	0.3831	1.0000	R_Superior_6-8_Transitional_Area
**Mean**					0.3690	0.3709	0.5678	
**4. Right DLPFC**								
264	Cingular-Opercular	36	41	30	0.3608	0.3625	0.0000	R_Area_46
266	Cingular-Opercular	29	50	22	0.3750	0.3770	0.0547	R_Area_9-46d
**Mean**					0.3679	0.3698	0.0274	
**5. Right Precentral**								
190	Cingular-Opercular	44	-2	51	0.3653	0.3667	0.0391	R_Frontal_Eye_Fields
191	Cingular-Opercular	47	3	37	0.3618	0.3625	0.0782	R_Premotor_Eye_Fields
192	Language	49	2	47	0.3996	0.4007	0.0078	R_Area_55b

253	Frontoparietal	40	18	36	0.3347	0.3357	0.0782	R_Area_IFJp
**Mean**					0.3654	0.3664	0.0508	
**6. Left IPL**								
148	Cingular-Opercular	-61	-36	36	0.3635	0.3653	0.6875	L_Area_PF_Complex
149	Frontoparietal	-50	-56	44	0.3767	0.3799	0.1094	L_Area_PFm_Complex
**Mean**					0.3701	0.3726	0.3985	

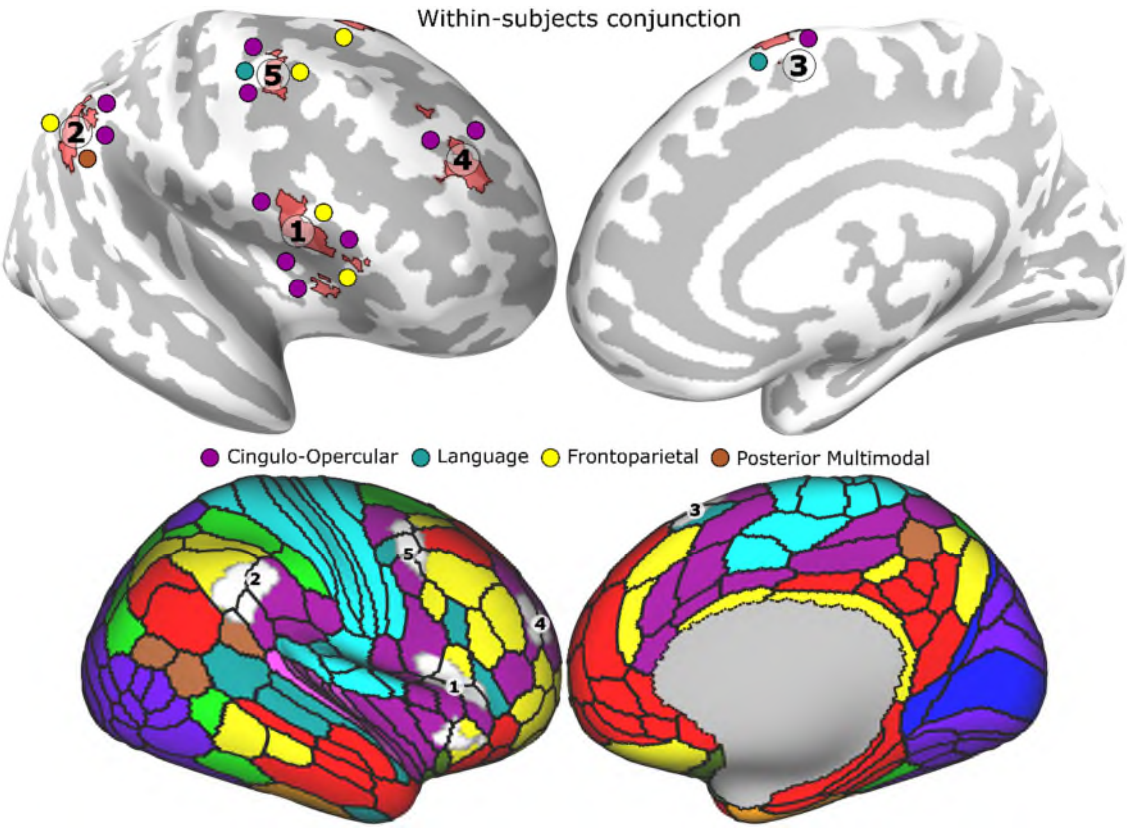

Supplementary Figure 1. Within-subjects conjunction clusters overlayed on the Cole-Anticevic brain-wide network partition (CAB-NP). The top panel shows our within-subjects conjunction clusters and estimated CAB-NP network assignments. The number of dots indicates how many parcels our cluster was assigned to within this network. Dots were placed manually and are for visualisation purposes only. **The bottom panel** shows coloured CAB-NP, our within-subjects conjunction clusters in white and foci of our clusters as the corresponding numbers. The foci were placed based on the MNI coordinates from the Table 1 of the main text. The conjunction map is available on the GitHub repository[5] (https://github.com/dcdace/Domain-general/).

Supplementary Table 2. Meta-analytic Stop > Go & No-Think > Think conjunction clusters (from Table 1 in the main text) and their estimated parcels, networks, centre X, Y, Z coordinates, global variability coefficients (GVC), between-network variability coefficients (BVC), network partition deviation numbers, and parcel descriptions.

Parcel	Network	X	Y	Z	GVC	BVC	deviation	Parcel description
**1. Right VLPFC & Insula**								
254	Frontoparietal	53	19	13	0.3469	0.3486	0.6563	R_Area_44
288	Cingular-Opercular	39	16	6	0.3717	0.3721	0.8907	R_Frontal_Opercular_Area_4
289	Cingular-Opercular	38	13	1	0.3663	0.3691	0.1172	R_Middle_Insular_Area
291	Frontoparietal	33	26	-4	0.3289	0.3299	0.3204	R_Anterior_Ventral_Insular_Area
349	Cingular-Opercular	38	28	4	0.3714	0.3724	0.0078	R_Area_Frontal_Opercular
**Mean**					0.3570	0.3584	0.3985	
Cingular-Opercular					0.3698	0.3712	0.3386	
Frontoparietal					0.3379	0.3393	0.4884	
**2. Right/Left SMA**								
206	Language	8	19	64	0.3794	0.3819	0.1954	R_Superior_Frontal_Language_Area
223	Cingular-Opercular	6	6	58	0.3928	0.3951	0.0625	R_Supplementary_And_Cingulate_Eye
224	Cingular-Opercular	20	7	66	0.3463	0.3477	0.5079	R_Area_6m_anterior
243	Frontoparietal	5	32	46	0.3692	0.3698	0.2344	R_Area_8BM
278	Frontoparietal	20	25	57	0.3812	0.3831	1.0000	R_Superior_6-8_Transitional_Area
**Mean**					0.3738	0.3755	0.4000	
**3. Left VLPFC & Insula**								
109	Cingular-Opercular	-37	11	3	0.3519	0.3552	0.2422	L_Middle_Insular_Area
111	Frontoparietal	-31	25	-3	0.3805	0.3847	0.3438	L_Anterior_Ventral_Insular_Area
**Mean**					0.3662	0.3700	0.2930	
**4. Right IPL**								
205	Cingular-Opercular	63	-37	27	0.3789	0.3818	0.1797	R_PeriSylvian_Language_Area
208	Posterior Multimodal	57	-45	22	0.3609	0.3620	0.3282	R_Superior_Temporal_Visual_Area
328	Cingular-Opercular	60	-30	38	0.3443	0.3446	0.0000	R_Area_PF_Complex
329	Frontoparietal	51	-50	45	0.3351	0.3361	0.2501	R_Area_PFm_Complex
**Mean**					0.3548	0.3561	0.1895	
**5. Right ACC**								
239	Cingular-Opercular	4	19	32	0.3716	0.3705	0.8047	R_Anterior_24_prime
240	Cingular-Opercular	9	15	39	0.3795	0.3783	0.5782	R_Area_p32_prime
243	Frontoparietal	5	32	46	0.3692	0.3698	0.2344	R_Area_8BM
359	Cingular-Opercular	9	29	30	0.3592	0.3595	0.9922	R_Area_anterior_32_prime
**Mean**					0.3699	0.3695	0.6524	
**6. Right DLPFC**								
264	Cingular-Opercular	36	41	30	0.3608	0.3625	0.0000	R_Area_46
266	Cingular-Opercular	29	50	22	0.3750	0.3770	0.0547	R_Area_9-46d
**Mean**					0.3679	0.3698	0.0274	
**7. Basal ganglia**								
**8. Left IPL**								
148	Cingular-Opercular	-61	-36	36	0.3635	0.3653	0.6875	L_Area_PF_Complex
149	Frontoparietal	-50	-56	44	0.3767	0.3799	0.1094	L_Area_PFm_Complex
**Mean**					0.3701	0.3726	0.3985	
**9. Right Precentral**								
190	Cingular-Opercular	44	-2	51	0.3653	0.3667	0.0391	R_Frontal_Eye_Fields
192	Language	49	2	47	0.3996	0.4007	0.0078	R_Area_55b
**Mean**					0.3825	0.3837	0.0235	
**10. Right SPL**								
297	Dorsal Attention	38	-38	44	0.3888	0.3899	0.9610	R_Anterior_IntraParietal_Area
324	Frontoparietal	42	-42	46	0.3960	0.4011	0.2500	R_Area_IntraParietal_2
**Mean**					0.3924	0.3955	0.6055	

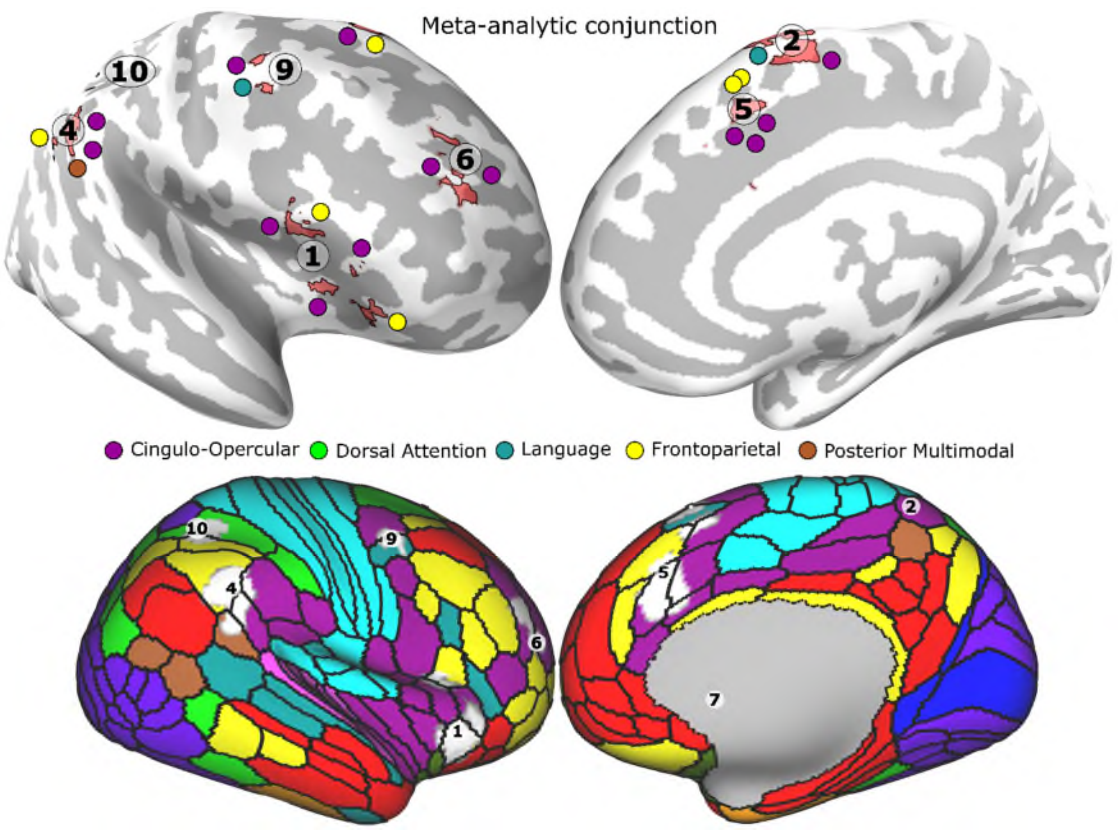

Supplementary Figure 2. Meta-analytic conjunction clusters overlayed on the Cole-Anticevic brain-wide network partition (CAB-NP). The top panel shows our meta-analytic conjunction clusters and estimated CAB-NP network assignments. The number of dots indicates how many parcels our cluster was assigned to within this network. Dots were placed manually and are for visualisation purposes only. **The bottom panel** shows coloured CAB-NP, our meta-analytic conjunction clusters in white and foci of our clusters as the corresponding numbers. The foci were placed based on the MNI coordinates from the Table 1 of the main text. The conjunction map is available on the GitHub repository[5] (https://github.com/dcdace/Domain-general/).

Supplementary Table 3. Within-subjects & meta-analytic conjunction clusters (from Table 1 in the main text) and their estimated parcels, networks, centre X, Y, Z coordinates, global variability coefficients (GVC), between-network variability coefficients (BVC), network partition deviation numbers, and parcel descriptions.

Parcel	Network	X	Y	Z	GVC	BVC	deviation	Parcel description
**1. Right VLPFC & Insula**								
254	Frontoparietal	53	19	13	0.3469	0.3486	0.6563	R_Area_44
288	Cingular-Opercular	39	16	6	0.3717	0.3721	0.8907	R_Frontal_Opercular_Area_4
289	Cingular-Opercular	38	13	1	0.3663	0.3691	0.1172	R_Middle_Insular_Area
291	Frontoparietal	33	26	-4	0.3289	0.3299	0.3204	R_Anterior_Ventral_Insular_Area
349	Cingular-Opercular	38	28	4	0.3714	0.3724	0.0078	R_Area_Frontal_Opercular
**Mean**					0.3570	0.3584	0.3985	
Cingular-Opercular					0.3698	0.3712	0.3386	
Frontoparietal					0.3379	0.3393	0.4884	
**2. Right IPL**								
205	Cingular-Opercular	63	-37	27	0.3789	0.3818	0.1797	R_PeriSylvian_Language_Area
208	Posterior Multimodal	57	-45	22	0.3609	0.3620	0.3282	R_Superior_Temporal_Visual_Area
328	Cingular-Opercular	60	-30	38	0.3443	0.3446	0.0000	R_Area_PF_Complex
329	Frontoparietal	51	-50	45	0.3351	0.3361	0.2501	R_Area_PFm_Complex
**Mean**					0.3548	0.3561	0.1895	
**3. Right SMA**								
206	Language	8	19	64	0.3794	0.3819	0.1954	R_Superior_Frontal_Language_Area
278	Frontoparietal	20	25	57	0.3812	0.3831	1.0000	R_Superior_6-8_Transitional_Area
224	Cingular-Opercular	20	7	66	0.3463	0.3477	0.5079	R_Area_6m_anterior
**Mean**					0.3690	0.3709	0.5678	
**4. Right DLPFC**								
264	Cingular-Opercular	36	41	30	0.3608	0.3625	0.0000	R_Area_46
266	Cingular-Opercular	29	50	22	0.3750	0.3770	0.0547	R_Area_9-46d
**Mean**					0.3679	0.3698	0.0274	
**5. Left IPL**								
148	Cingular-Opercular	-61	-36	36	0.3635	0.3653	0.6875	L_Area_PF_Complex
149	Frontoparietal	-50	-56	44	0.3767	0.3799	0.1094	L_Area_PFm_Complex
**Mean**					0.3701	0.3726	0.3985	
**6. Right Precentral**								
190	Cingular-Opercular	44	-2	51	0.3653	0.3667	0.0391	R_Frontal_Eye_Fields
192	Language	49	2	47	0.3996	0.4007	0.0078	R_Area_55b
**Mean**					0.3825	0.3837	0.0235	

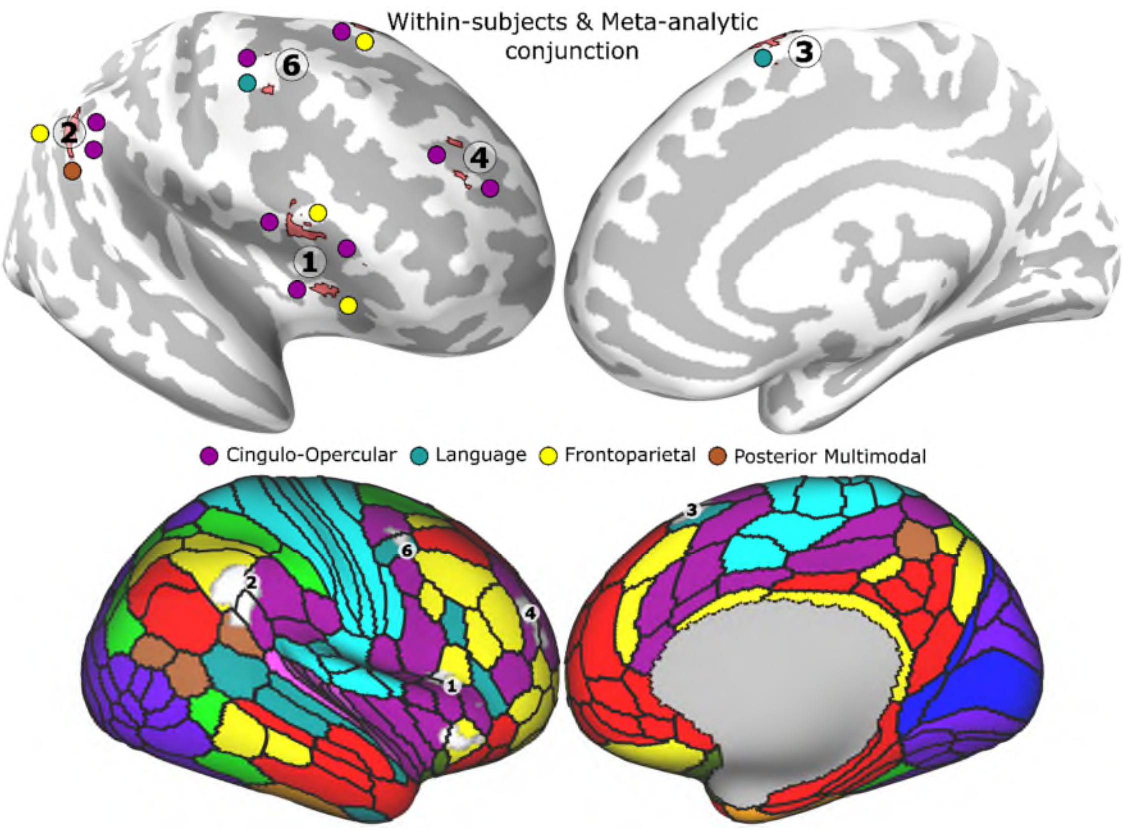

Supplementary Figure 3. Within-subjects & meta-analytic conjunction clusters overlayed on the Cole-Anticevic brain-wide network partition (CAB-NP). The top panel shows our within-subjects & meta-analytic conjunction results clusters and estimated CAB-NP network assignments. The number of dots indicates how many parcels our cluster was assigned to within this network. Dots were placed manually and are for visualisation purposes only. **The bottom panel** shows coloured CAB-NP, our within-subjects & meta-analytic conjunction results clusters in white and foci of our clusters as the corresponding numbers. The foci were placed based on the MNI coordinates from the Table 1 of the main text. The conjunction map is available on the GitHub repository[5] (https://github.com/dcdace/Domain-general/).

Supplementary Note 2: MVPA results accounting for ROI size differences

In the paper, we do not statistically compare the classification accuracies between the ROIs. We approach the existence of the domain-general and domain-specific components as a Yes/No question, which is sufficient for testing our hypotheses. Comparisons of classification accuracies across ROIs should be interpreted with caution[6]. Factors such as the number of voxels (the dimensionality difference), the haemodynamic response efficiency, and the signal-to-noise affect the classification accuracy and limit the comparison of accuracies between different ROIs. Nevertheless, as readers might be interested in the domain-general and domain-specific component differences across ROIs, we performed another version of the classification analysis. In this version, we account for the ROI size differences (the one factor we can account for). To account for the ROI size differences, in our random subset selection, we kept the size of each subset constant across the 4 ROIs. That way, within each subject, for all four ROIs, the classifier had an equal number of dimensions.

The results of this adjusted analysis are presented in Supplementary Figure 4 below. For the domain-general component, one-way ANOVA revealed significant difference between the four ROIs: $F_{3,69} = 4.976$, $p = 0.003$. However, there was no significant difference for the domain-specific component: $F_{3, 69} = 1.703$, $p = 0.174$.

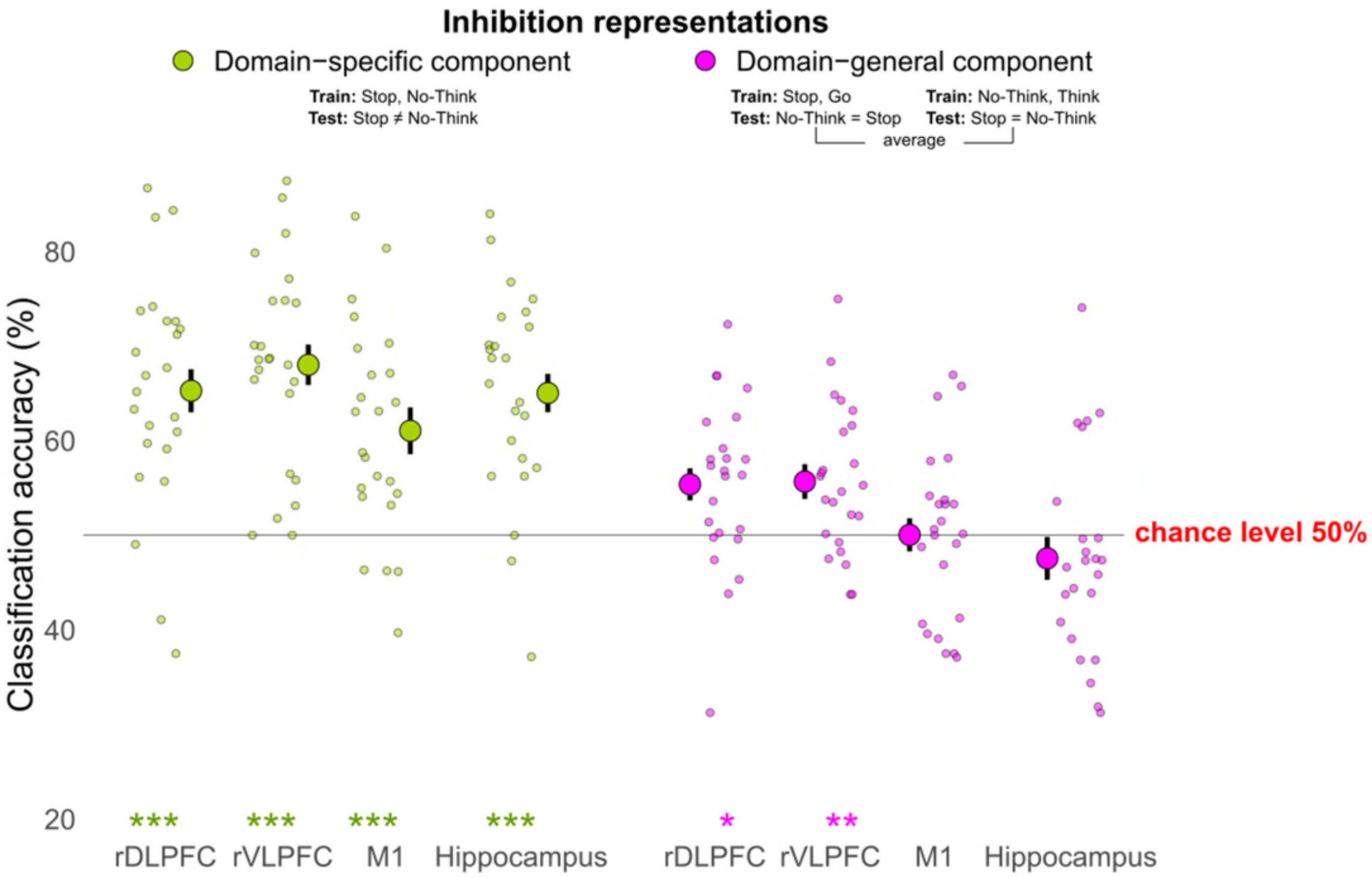

Supplementary Figure 4. MVPA results accounting for ROI size differences. ***p < 0.001; **p < 0.01; *p < 0.05. Error bars represent within-subject standard error. Source data are provided as a Source Data file.

Supplementary Note 3: Domain-general classification for all meta-analytic conjunction ROIs

To choose source regions for a control DCM analysis, we performed the domain-general classification analysis on all ten meta-analytic conjunction regions (see Table 1b and Table S2). Apart from rDLPFC and rVLPFC, only the right and left inferior parietal lobule (IPL) exhibited significant domain-general component (see Supplementary Figure 5). Note that here, as in our main classification analyses, we do not account for size differences across ROIs.

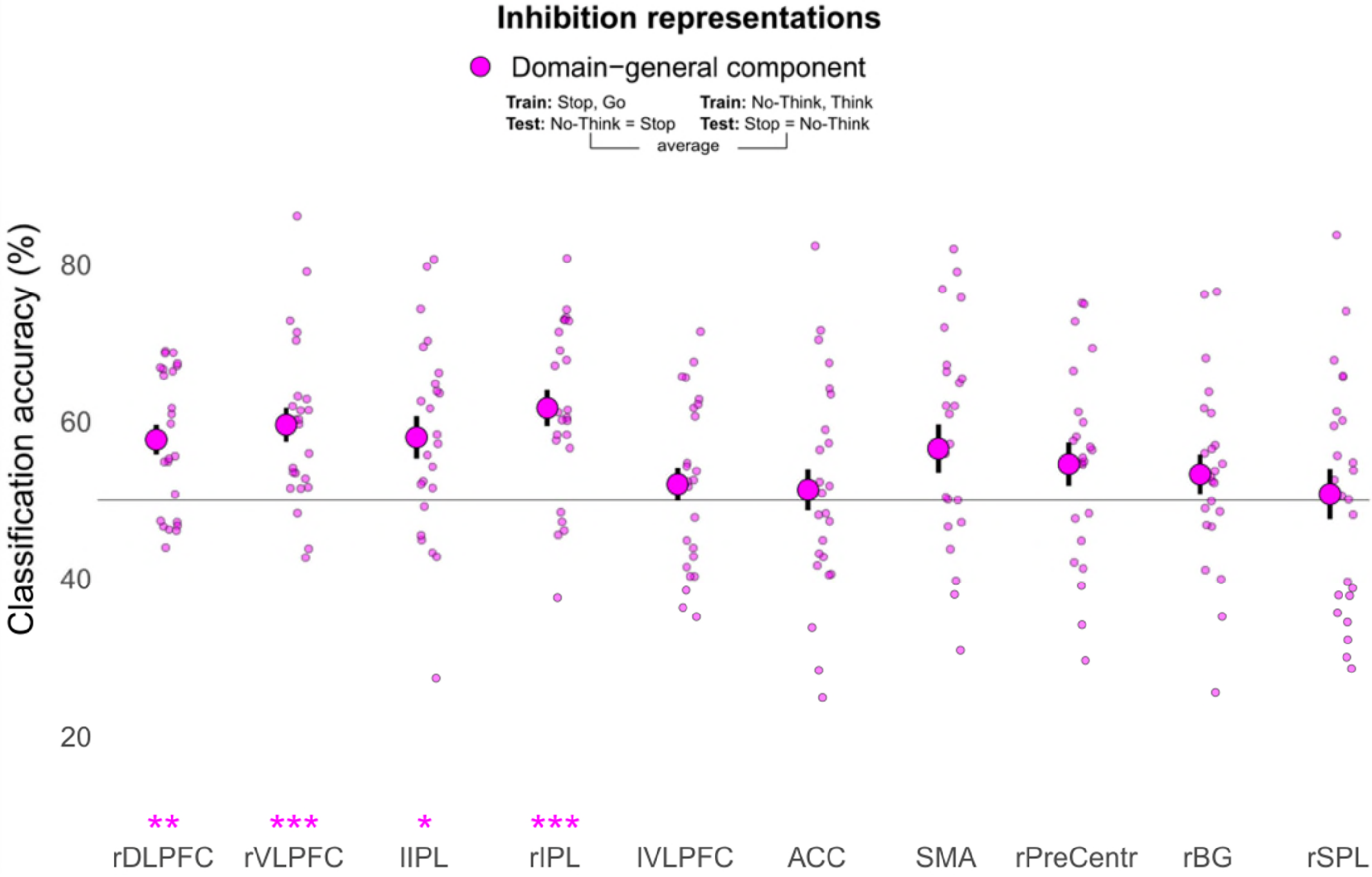

Supplementary Figure 5. Domain-general classification results for all meta-analytic conjunction ROIs (see Table 1b and Table S2). The classification results are Bonferroni corrected for 10 comparisons. ***p < 0.001; **p < 0.01; *p < 0.05. Error bars represent within-subject standard error. Source data are provided as a Source Data file.

Supplementary Note 4: Control DCM analysis

We considered the possibility that goal-dependent top-down connectivity with the hippocampus and M1 would arise for any brain region robustly activated by both action and retrieval stopping, and not just the rDLPFC and rVLPFC. To test this possibility, we modified our DCM analysis by replacing the rDLPFC and rVLPFC nodes with two other regions from our meta-analytic conjunction analysis as sources of control. To choose the control regions, we performed our domain-general classification analysis on all ten meta-analytic conjunction regions (see the previous section). Apart from rDLPFC and rVLPFC, only the right and left inferior parietal lobule (IPL) exhibited significant domain-general component (see Supplementary Figure 5).

We replaced rDLPFC and rVLPFC with the right IPL and left IPL in our original model space of 73 models (see Figure 7) to see if there was compelling evidence for a model in which this pair of regions behaved like our prefrontal regions. The exceedance probabilities of our original results and the control results are displayed in Supplementary Figure 6. As shown in the left panel, there is a clear winning model in the rDLPFC/rVLPFC analysis (exceedance probability of .92). In contrast, the analysis using the IPL regions (right panel) does not yield a model that provides a compelling account of the data (the highest exceedance probability is .25). The control DCM results suggest that the targeted inhibitory control signal is unlikely to originate from IPL and that our emphasis on the right lateral PFC is justified.

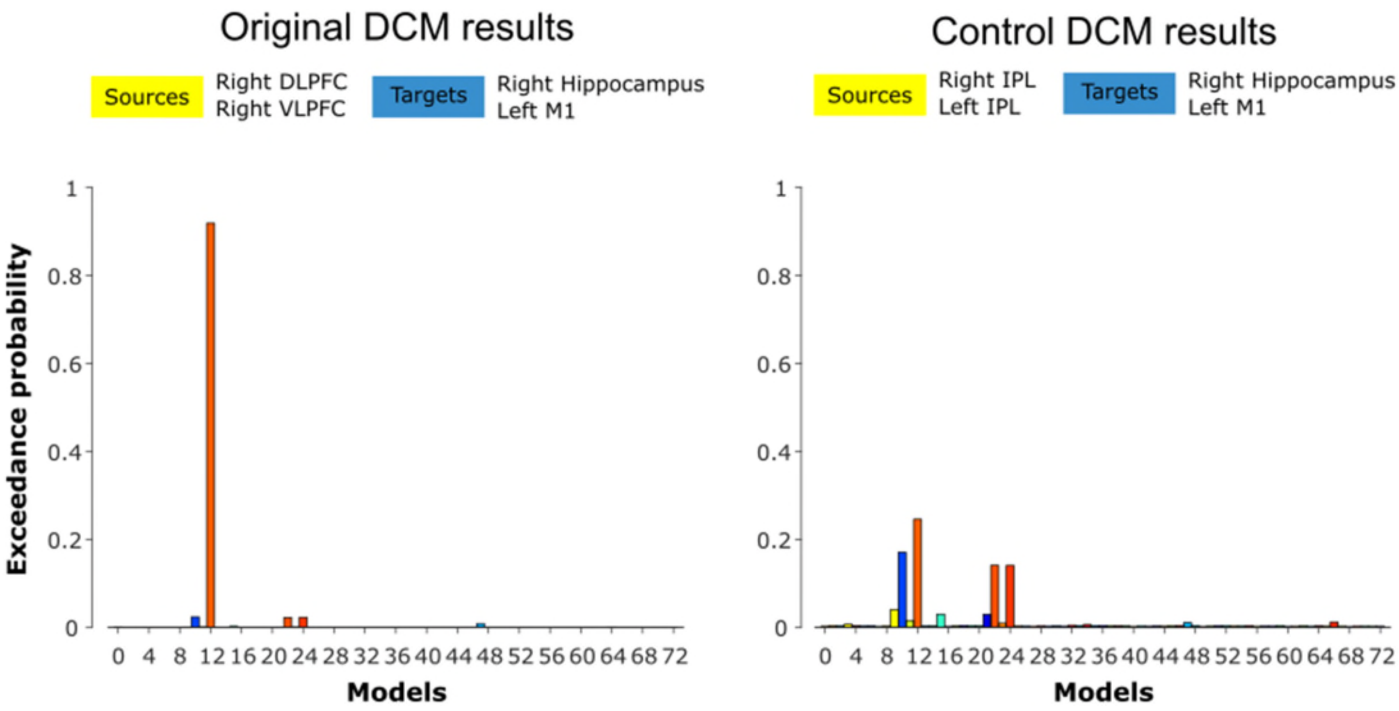

Supplementary Figure 6. Original and control DCM results. Source data are provided as a Source Data file.

Supplementary Note 5: MVPA confusion matrices

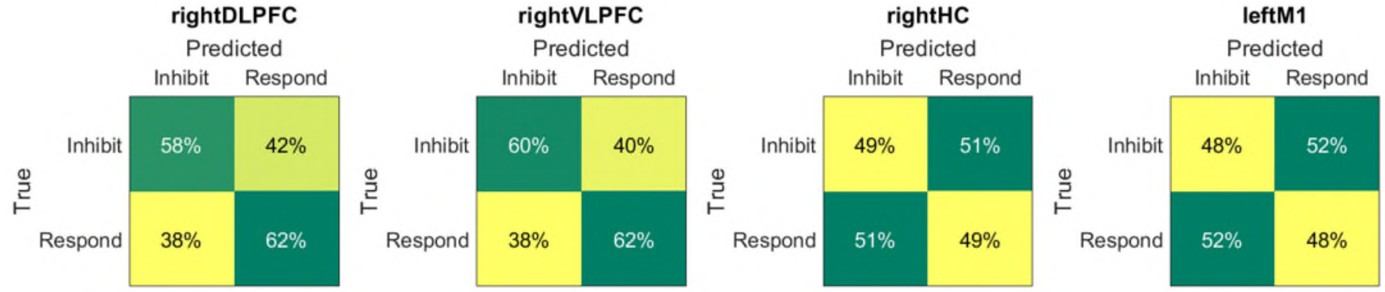

Supplementary Figure 7. Domain-general classification confusion matrices for each ROI.

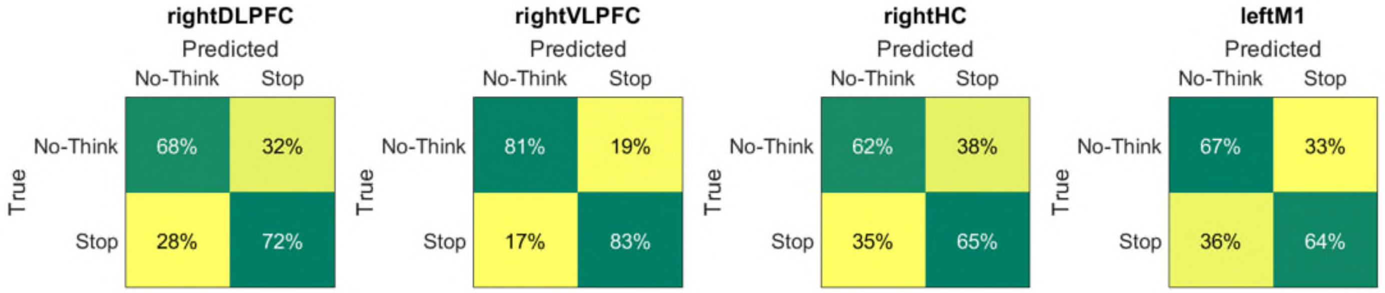

Supplementary Figure 8. Domain-specific classification confusion matrices for each ROI.

Supplementary References

1. Ji, J. L. *et al.* Mapping the human brain's cortical-subcortical functional network organization. *Neuroimage* **185**, 35–57 (2019).

2. Glasser, M. F. *et al.* A multi-modal parcellation of human cerebral cortex. *Nature* **536**, 171–178 (2016).

3. Marcus, D. S. *et al.* Informatics and data mining tools and strategies for the human connectome project. *Front. Neuroinform.* **5**, 1–12 (2011).

4. Cocuzza, C. V, Ito, T., Schultz, D., Bassett, D. S. & Cole, M. W. Flexible coordinator and switcher hubs for adaptive task control. *J. Neurosci.* JN-RM-2559-19 (2020). doi:10.1523/JNEUROSCI.2559-19.2020

5. Apšvalka, D., Ferreira, C. S., Schmitz, T. W., Rowe, J. B. & Anderson, M. C. Dynamic targeting enables domain-general inhibitory control over action and thought by the prefrontal cortex (data & code) [Data set]. *Zenodo* (2021). doi:10.5281/zenodo.5732892

6. Haynes, J.-D. A Primer on Pattern-Based Approaches to fMRI: Principles, Pitfalls, and Perspectives. *Neuron* **87**, 257–270 (2015).
