# Supplementary material for: Dynamic targeting enables domain-general inhibitory control over action and thought by the prefrontal cortex
Source: Nat Commun. 2022 Jan 12;13:274. doi: 10.1038/s41467-021-27926-w (PMC8755760; doi:10.1038/s41467-021-27926-w)
Supplement: Supplementary file 3 — Reporting summary [file 41467_2021_27926_MOESM3_ESM.pdf]

nature research

Corresponding author(s): Dace Apšvalka; Michael C, Anderson

Last updated by author(s): Dec 17, 2021

Reporting Summary

Nature Research wishes to improve the reproducibility of the work that we publish. This form provides structure for consistency and transparency in reporting. For further information on Nature Research policies, see our Editorial Policies and the Editorial Policy Checklist.

Statistics

For all statistical analyses, confirm that the following items are present in the figure legend, table legend, main text, or Methods section.

n/a	Confirmed	
[ ]	[x]	The exact sample size (*n*) for each experimental group/condition, given as a discrete number and unit of measurement
[ ]	[x]	A statement on whether measurements were taken from distinct samples or whether the same sample was measured repeatedly
[ ]	[x]	The statistical test(s) used AND whether they are one- or two-sided<br>*Only common tests should be described solely by name; describe more complex techniques in the Methods section.*
[x]	[ ]	A description of all covariates tested
[ ]	[x]	A description of any assumptions or corrections, such as tests of normality and adjustment for multiple comparisons
[ ]	[x]	A full description of the statistical parameters including central tendency (e.g. means) or other basic estimates (e.g. regression coefficient) AND variation (e.g. standard deviation) or associated estimates of uncertainty (e.g. confidence intervals)
[ ]	[x]	For null hypothesis testing, the test statistic (e.g. *F*, *t*, *r*) with confidence intervals, effect sizes, degrees of freedom and *P* value noted<br>*Give P values as exact values whenever suitable.*
[x]	[ ]	For Bayesian analysis, information on the choice of priors and Markov chain Monte Carlo settings
[ ]	[x]	For hierarchical and complex designs, identification of the appropriate level for tests and full reporting of outcomes
[ ]	[x]	Estimates of effect sizes (e.g. Cohen's *d*, Pearson's *r*), indicating how they were calculated

Our web collection on statistics for biologists contains articles on many of the points above.

Software and code

Policy information about availability of computer code

Data collection	Presentation software (Neurobehavioral Systems, Albany, CA, USA); optseq2, http://surfer.nmr.mgh.harvard.edu/optseq; 3-Tesla Siemens Tim Trio MRI scanner (Siemens, Erlangen, Germany)
Data analysis	R (v4, 2020-04-24) ; SPM v12r7487; MATLAB vR2012a; GingerALE v3.0.2 ; Connectome Workbench; ITK-SNAP v3.8.0; for fMRI image display: FreeSurfer v7, and PySurfer v0.10.0 and Nilearn v0.8.1, Python packages.<br>Custom code: https://github.com/dcdace/Domain-general/

For manuscripts utilizing custom algorithms or software that are central to the research but not yet described in published literature, software must be made available to editors and reviewers. We strongly encourage code deposition in a community repository (e.g. GitHub). See the Nature Research guidelines for submitting code & software for further information.

Data

Policy information about availability of data

All manuscripts must include a data availability statement. This statement should provide the following information, where applicable:

- Accession codes, unique identifiers, or web links for publicly available datasets
- A list of figures that have associated raw data
- A description of any restrictions on data availability

The behavioural and ROI data have been deposited on the GitHub repository https://github.com/dcdace/Domain-general/

Field-specific reporting

Please select the one below that is the best fit for your research. If you are not sure, read the appropriate sections before making your selection.

- [ ] Life sciences
- [x] Behavioural & social sciences
- [ ] Ecological, evolutionary & environmental sciences

For a reference copy of the document with all sections, see nature.com/documents/nr-reporting-summary-flat.pdf

Behavioural & social sciences study design

All studies must disclose on these points even when the disclosure is negative.

Study description	This is a quantitative, experimental, within-subjects study assessing behavioural and neural responses to stopping actions and stopping thoughts.
Research sample	Participants were a representative sample from Cambridge (UK) community. The final sample comprised 24 participants (7 males, 17 females), 19-36 years old (M = 24.67 years, SD = 4.31). The chosen study sample is believed to be representative of healthy young adults.
Sampling strategy	A convenience sampling procedure was used.<br>No formal sample-size calculation was performed. Our final sample of N=24 is comparable to previous published fMRI studies of similar designs for testing medium to large effects.
Data collection	We presented stimuli and recorded responses with Presentation software (Neurobehavioral Systems, Albany, CA, USA). The stimuli were projected onto a screen that participants viewed during the MRI scan using a mirror attached to the head coil. Participants responded by pressing one of the two buttons (left or right) with a dominant (right) hand on a button box. The fMRI acquisition was performed with 3-Tesla Siemens Tim Trio MRI scanner (Siemens, Erlangen, Germany) fitted with a 32-channel head coil.<br>A research assistant and MR technician were present during data collection. Participants were aware of the experimental conditions but not the study hypothesis.<br>The experimenters were aware of the conditions and hypotheses, however all instructions were provided on screen with no interaction required by the experimenter during the task.
Timing	May - July 2013
Data exclusions	From the initial N=30, 5 subjects were excluded due to not reaching required learning criterion on the Think/No-Think task, 1 subject fell asleep during fMRI acquisition. From the N=24 included in the final sample, 1 bi-variate outlier was removed from the correlation analysis and the behavioural partial least squares analysis. Outliers were determined using box plot method, which relies on the interquartile range.
Non-participation	One participant dropped out due to falling asleep during fMRI acquisition.
Randomization	Participants were not allocated into experimental groups.

Reporting for specific materials, systems and methods

We require information from authors about some types of materials, experimental systems and methods used in many studies. Here, indicate whether each material, system or method listed is relevant to your study. If you are not sure if a list item applies to your research, read the appropriate section before selecting a response.

Materials & experimental systems

n/a	Involved in the study	
x		Antibodies
x		Eukaryotic cell lines
x		Palaeontology and archaeology
x		Animals and other organisms
	x	Human research participants
x		Clinical data
x		Dual use research of concern

Methods

n/a	Involved in the study	
x		ChIP-seq
x		Flow cytometry
	x	MRI-based neuroimaging

Human research participants

Policy information about studies involving human research participants

Population characteristics	See above.
Recruitment	Participants were recruited from the community via mailing lists, flyers, word of mouth, web advertising and volunteer participant pool. No selection biases were expected.
Ethics oversight	The Cambridge Psychology Research Ethics Committee approved the project.

Note that full information on the approval of the study protocol must also be provided in the manuscript.

Magnetic resonance imaging

Experimental design

Design type	Event-related
Design specifications	8 fMRI scanning runs in a single session. During each fMRI run, participants performed 8 blocks of the Think/No-Think task interleaved with 8 blocks of the Stop-signal task. All blocks lasted 30 s. To minimize carry-over effects, we interspersed 4 s rest periods between the task blocks.<br>Four of the Stop-signal task blocks contained Go trials only. We did not use these blocks in this report. Each of the other four Stop-signal blocks contained 12 trials, yielding 384 trials in total (8 runs * 4 blocks per run * 12 trials per block).<br>Each of the Think/No-Think blocks contained 6 trials, starting with a filler item as a Think trial followed by 5 Think or No-Think items in a pseudo-random order. Within each fMRI run, participants saw all 20 critical Think and 20 critical No-Think items once. Thus, across the 8 runs, participants recalled or suppressed each memory item 8 times.
Behavioral performance measures	Stop-signal task variables recorded during the fMRI acquisition: button pressed or not; which button pressed (1 or 2); correct/incorrect response for Go trials; RT for Go trials; RT for failed Stop trials.<br>We calculated stop-signal reaction time, probability of Go omissions, probability of choice errors on Go trials, probability of responding to Stop trials, mean stop-signal delay of all Stop trials, mean correct Go RT, and mean failed Stop RT.<br>Probability of Go omissions and choice errors on Go trials were negligible indicating that participants were performing the task as required. We also compared RTs of all Go trials against RTs of failed Stop trials to test the assumption of an independent race between a go and a stop runner; that was confirmed for all but one participant.<br>For the Think/No-Think task, we did not record any variables during the fMRI acquisition. But in the middle of the scanning session (after the 4th block), we administered a diagnostic questionnaire to ensure that participants closely followed the instructions of the Think/No-Think task. After the scanning, the task performance was assessed by verifying that our Think/No-Think task had induced forgetting of suppressed items.

Acquisition

Imaging type(s)	Functional and structural.
Field strength	3T
Sequence & imaging parameters	We acquired functional brain volumes using a gradient-echo, T2*-weighted echoplanar pulse sequence (TR = 2000 ms, TE = 30 ms, flip angle = 90°, 32 axial slices, descending slice acquisition, voxel resolution = 3 mm^3, 0.75 mm interslice gap).<br>We acquired an anatomical reference for each participant, a high-resolution whole-brain 3D T1-weighted magnetization-prepared rapid gradient echo (MP-RAGE) image (TR = 2250 ms, TE =2.99 ms, flip angle = 9°, field of view = 256 x 240 x 192 mm, voxel resolution = 1 mm^3).
Area of acquisition	Whole brain

Diffusion MRI ☐ Used ☒ Not used

Preprocessing

Preprocessing software	SPM 12
Normalization	We submitted the segmented images for each participant to the DARTEL procedure to create a group-specific anatomical template which optimises inter-participant alignment. The DARTEL procedure alternates between computing a group template and warping an individual's tissue probability maps into alignment with this template and ultimately creates an individual flow field of each participant. Subsequently, the procedure transformed the group template into MNI-152 space. Finally, we applied the MNI transformation and smoothing with an 8 mm full-width-at-half-maximum (FWHM) Gaussian kernel to the functional images for the whole-brain voxel-wise analysis.<br>ROI, DCM, and MPVA analyses were performed in subjects' native space.
Normalization template	MNI-152

Noise and artifact removal	SPM 12 default motion correction and slice-timing correction was applied. In the GLM, motion parameters accounting for 6 degrees of freedom were regressed out.
Volume censoring	None

Statistical modeling & inference

Model type and settings	Univariate whole-brain analysis; Meta-analytical whole-brain analysis; Behavioural partial least squares (PLS) analysis; ROI analysis; Multi-voxel pattern analysis; Dynamic causal modelling (DCM) analysis.<br>All group-level analysis were random-effect.
Effect(s) tested	* Univariate whole-brain analysis.<br>First-level, subject specific, t-tests for Stop, Go, No-Think, and Think conditions, and the main effect of Inhibit [Stop, No-Think] > Respond [Go, Think].<br>Second level, random-effects, repeated-measures ANOVA; conjunction analysis of Stop > Go & No-Think > Think contrasts.<br><br>* Meta-analytical whole-brain analysis.<br>First, separate meta-analyses of Stop > Go, No-Think > Think, and their pooled data. Then, we submitted the obtained thresholded ALE maps from the three individual meta-analyses to a meta-analytic contrast analysis, which produced the conjunction of the Stop > Go & No-Think > Think contrasts.<br><br>* Behavioural partial least squares (PLS) analysis.<br>Within the meta-analytic conjunction mask, we identified voxels where the BOLD signal from the main effect of Inhibit > Respond contrast depicted the largest joint covariance with the stop-signal reaction time (SSRT) and suppression-induced forgetting (SIF) scores.<br><br>* ROI analysis.<br>Repeated-measures ANOVA; post-hoc pairwise comparisons.<br><br>* Multi-voxel pattern analysis.<br>Linear discriminant analysis (LDA) to classify voxel activity patterns. At the group level, for each ROI, we performed one-tailed t-tests to assess the statistical significance of classification accuracy being above the 50% chance level.<br><br>* Dynamic causal modelling (DCM) analysis.<br>We compared 73 models using Bayesian model selection.

Specify type of analysis: ☐ Whole brain ☐ ROI-based ☒ Both

Anatomical location(s)	ROIs: the right dorsolateral prefrontal cortex (rDLPFC), the right ventrolateral prefrontal cortex (rVLPFC), the right hippocampus, and the left M1.<br>We obtained the rDLPFC and rVLPFC ROIs, centred at MNI coordinates 35, 45, 24 and 44, 21, -1, respectively, from an independent meta-analytic conjunction analysis (described methods). We defined the M1 ROI (centred at MNI coordinates -33, -22, 46) from a group analysis (N = 30) of an independent M1 localiser study on different participants (Button Press > View contrast). We mapped the rDLPFC, rVLPFC, and M1 ROIs from the MNI space to participants' native space. We manually traced the hippocampal ROIs in native space for each participant, using ITK-SNAP (www.itksnap.org; Yushkevich et al., 2006) and following established anatomical guidelines (Duvernoy et al., 2013; Pruessner et al., 2000).

Statistic type for inference<br>(See Eklund et al. 2016)	Univariate: voxel-wise.<br>Meta-analysis:<br>* cluster-wise for the 3 separate meta-analyses (cluster-level FWE corrected inferencel; p < 0.05, cluster-forming threshold uncorrected p < 0.001, threshold permutations = 1000).<br>* voxel-wise for the meta-analytic conjunction (voxel-wise uncorrected p < 0.001, with the p-value permutations of 10,000 iterations, and the minimum cluster volume of 200 mm^3).
Correction	* Univariate conjunction analysis was thresholded at p < 0.05 FDR corrected for whole-brain multiple comparisons.<br>* Meta-analytic conjunction was thresholded at voxel-wise uncorrected p < 0.001, with the p-value permutations of 10,000 iterations, and the minimum cluster volume of 200 mm^3.<br>* ROI post-hoc comparisons were Bonferroni corrected.<br>* Multi-variate tests were Bonferroni corrected.

Models & analysis

n/a	Involved in the study	
☐	☒	Functional and/or effective connectivity
☒	☐	Graph analysis
☐	☒	Multivariate modeling or predictive analysis

Functional and/or effective connectivity	Dynamic causal modelling (DCM), comparing 73 models using Bayesian model selection.
Multivariate modeling and predictive analysis	We used linear discriminant analysis (LDA) to classify voxel activity patterns within four ROIs: rDLPFC, rVLPFC, right hippocampus, and left M1.<br>To increase the reliability of pattern classification accuracy, we used a random subset approach (Diedrichsen et al., 2013). Specifically, for each ROI separately, we created up to 2000 unique subsets of randomly drawn 90% of ROI voxels. We then applied the LDA on each subset and averaged the subset results to obtain the final classification accuracy for each ROI.<br>We performed two types of pattern classification to identify domain-general and domain-specific components within each ROI.<br><br>For the domain-general component, we performed a cross-task classification. We trained the LDA classifier to distinguish Inhibit from Respond conditions in one modality (e.g. No-Think from Think) and tested whether the trained classifier could distinguish Inhibit from Respond in the other modality (e.g. Stop from Go). Both training and testing data consisted of two (conditions) by eight (runs) activation estimates for a set of voxels (e.g. 13 x 16 matrix for a set of 13 voxels). For training and testing sets separately, for each voxel, we z-scored the activity patterns across the 16 activation estimates setting the mean activity within each voxel to zero. This way, each voxel represented only the relative contribution of Inhibit vs Respond conditions within the Think/No-Think and Stop-signal tasks. For each ROI subset, we performed the LDA twice. The first classifier trained to discriminate Think from No-Think and returned the accuracy of distinguishing Stop from Go; the second classifier trained to discriminate Stop from Go and returned the accuracy of distinguishing Think from No-Think. The final score was the average classification accuracy of all subsets and the two classification variants (up to 2000 x 2) per ROI and subject.<br><br>For the domain-specific component, we trained and tested the LDA classifier to distinguish No-Think from Stop conditions. The input data consisted of two (conditions) by eight (runs) activation estimates for a set of voxels. We z-scored the activity patterns across voxels for each event of interest. Thus, the mean ROI activity for each event was zero, and each voxel represented only its relative contribution to the given event. That way, we accounted for the univariate intensity differences between No-Think and Stop conditions. For each ROI subset, we performed leave-one-run out cross-validated LDA and averaged the classification accuracies across the eight cross-validation folds. The final score was the average classification accuracy of all subsets and cross-validation folds (up to 2000 x 8) per ROI and subject.<br><br>At the group level, for each ROI, we performed one-tailed t-tests to assess the statistical significance of classification accuracy being above the 50% chance level. All tests were Bonferroni corrected for the number of ROIs.
